# A Clinical Decision-Making Algorithm for Botulinum Toxin Use in Temporomandibular Disorders and Bruxism

**DOI:** 10.3390/jcm15020755

**Published:** 2026-01-16

**Authors:** Anna N. Scheiwiler, Muhammed Ilhan, Oliver V. Waldvogel, Lukas B. Seifert, Florian M. Thieringer, Britt-Isabelle Berg

**Affiliations:** 1 Department of Oral and Cranio-Maxillofacial Surgery, University Hospital Basel, 4031 Basel, Switzerland; annanoemi.scheiwiler@stud.unibas.ch (A.N.S.);; 2 Department of Pediatric Oral Health and Orthodontics, University Center for Dental Medicine (UZB), University of Basel, 4058 Basel, Switzerland; 3 Medical Additive Manufacturing Research Group (Swiss MAM), Department of Biomedical Engineering, University of Basel, 4123 Allschwil, Switzerland; 4 Faculty of Medicine, University Hospital Basel, 4031 Basel, Switzerland

**Keywords:** botulinum toxin (BTX), temporomandibular disorders (TMD), bruxism, clinical decision algorithm, masseter Hypertrophy, dental decision support, orofacial pain

## Abstract

**Background:** Temporomandibular disorders (TMD) and bruxism are prevalent conditions managed by dentists. However, treatment choices—especially concerning botulinum toxin (BTX)—often lack consistency. This study aimed to develop and assess a structured clinical decision-making algorithm for BTX use in patients with TMD and bruxism. **Methods:** A treatment algorithm was designed through a qualitative analysis of the literature and aligned with German S3 guidelines. A total of 227 dentists assessed three clinical case vignettes reflecting routine clinical practice. Each vignette was evaluated first without and subsequently with the algorithm, focusing on typical indications for botulinum toxin treatment. Data were collected via online survey (SurveyMonkey) and analyzed using Microsoft Excel. Participants were stratified by gender and clinical experience (≤5 years vs. >5 years). **Results:** Of the 227 dentists contacted, 103 responded, and 56 completed the survey (57.1% male; mean age: 34.5 ± 10.6 years). BTX decision accuracy significantly improved for Case 1 (62.5% → 87.5%, *p* = 0.0013) and Case 2 (14.3% → 87.5%, *p* < 0.0001), but not for Case 3 (44.6% → 46.4%, *p* = 1.000). Confidence increased, and uncertainty decreased, particularly among less experienced dentists. The algorithm also significantly influenced both first- and second-line treatment choices, aligning them more closely with guideline-based therapy. Usefulness was confirmed by 78.6% of respondents, with no significant differences based on gender or experience. **Conclusions:** The proposed algorithm significantly improved diagnostic accuracy, treatment consistency, and confidence in the use of BTX for TMD and bruxism. It facilitates evidence-based, experience-independent decision-making and potentially represents a useful clinical tool in dental practice.

## 1. Introduction

Craniomandibular dysfunction (CMD), often referred to as temporomandibular disorders (TMD), encompasses a spectrum of conditions affecting the temporomandibular joint (TMJ) and the masticatory muscles. Terminology varies regionally: in Switzerland, these conditions are referred to as myoarthropathic complaints (MAP), in Germany CMD, and in English-speaking countries TMD is the predominant term [1,2,3]. To maintain consistency and readability, we use the term TMD throughout this manuscript.

The literature shows considerable variability in the reported prevalence of TMD. Recent meta-analyses indicate a global prevalence of TMD of approximately 30–34%, with notable geographical variability [4,5]. Given that nearly one-third of the population is already affected, the prevalence is projected to rise further by 2050, potentially developing into a significant public health concern. TMD may therefore represent a silent burden that has not yet received sufficient attention from clinicians, the community, or researchers [4,5].

Valesan et al. [6] aimed to summarize different assessment criteria and outcomes, reporting a TMD prevalence of 31.1% among adults and the elderly. Disc displacements were found in 19.1% of cases, and degenerative joint diseases in 9.8% [6]. However, the authors suggest that the true prevalence may be higher, as many patients with disc displacements remain asymptomatic and therefore go undiagnosed. Arthralgia prevalence is reported in 7% of the population [6]. TMD affects a broad age range, with the highest diagnosis rates occurring in early adulthood. Associated pain can diminish quality of life, prompt treatment, and lead to increased healthcare costs [7]. Up to 93% of individuals report experiencing at least one TMD symptom [8]. Bruxism has a prevalence of 22.1–31% during wakefulness and 12.8% ± 3.1% during sleep [9,10]. It affects both the TMJ and masticatory muscles [2]. Therefore, bruxism may contribute to TMD. Reported co-occurrence rates between bruxism and TMD vary widely. A recent meta-analysis estimated the global co-occurrence of bruxism and temporomandibular disorders at 17%, with marked geographic variation: approximately 70% in North America, 24% in South America, 14% in Europe, and 9% in Asia. Furthermore, the mean prevalence of TMD among individuals with bruxism was 63.5%, ranging from 98.3% in North America to 53.9% in Asia [11,12]. In patients with idiopathic inflammatory myopathies (IIMs), symptoms such as myalgia, myositis, and tenderness in the masticatory muscles on palpation are common and may be associated with TMD. Crincoli et al. [8] found that individuals with IIMs report more frequent complaints from the masticatory muscles than control subjects. Understanding the anatomy and function of the TMJ and masticatory muscles is essential for the targeted use of botulinum toxin in treating these disorders.

One of the most common signs of TMD is a clicking or snapping sound during jaw movements. This occurs when the condyle and disc move out of sync, causing the condyle to jump over the disc during motion. In more severe cases, this can result in a locking episode, in which disc displacement obstructs condylar movement [2,3]. Myofascial pain, typically caused by muscle overuse, is also frequently observed in TMD. Palpation usually reveals tenderness, and pain may radiate to the ears, head, and neck [2,3].

Myofascial pain is often misdiagnosed. In chronic cases, the primary muscular involvement is frequently overlooked, leading to inappropriate treatment; patients may receive therapy for depression or neuropathic disorders instead. When psychological factors are present, the condition is sometimes dismissed as psychosomatic. However, myofascial pain is a genuine physical disorder that requires targeted management [13]. It is characterized by persistent, localized pain, muscle tenderness, and restricted movement and is responsible for approximately 54.6% of headache and neck pain cases [13]. Trigger points in the masticatory muscles are commonly responsible for referred pain that extends beyond the affected area. Bruxism-induced overactivation of these muscles can further exacerbate the pain cycle [1,2,3].

Bruxism is defined as excessive jaw muscle activity, including clenching, grinding, and mandibular movements without occlusal contact [10]. In 2018, the classification of bruxism was refined to distinguish between sleep bruxism (SB), which occurs involuntarily during sleep, and awake bruxism (AB), which typically occurs while the individual is awake, often in response to stress or concentration [10]. Bruxism may also be categorized as primary (idiopathic) or secondary, when it is associated with factors such as sleep disorders, medication side effects, substance use, or neurological conditions [10]. The muscle contraction patterns further classify bruxism into tonic bruxism, characterized by sustained contractions lasting more than two seconds, phasic bruxism, involving repetitive contractions lasting between 0.25 and 2 s, and mixed forms, a combination of both, are also recognized [10].

Masseter hypertrophy, first described by Legg in 1880, presents as a progressive, painless facial swelling that may lead to functional impairments, including restricted mouth opening, cramping, vestibular narrowing due to muscle expansion, and TMJ clicking [14]. Although typically not associated with pain, hypertrophied masseters may contribute to TMJ discomfort and parotid gland swelling [14]. Therapeutic options for masseter hypertrophy include surgical reduction, conservative management through orthodontics and occlusal adjustments, and botulinum toxin injections as an off-label intervention [1,14,15]. Botulinum toxin, a neurotoxin produced by *Clostridium botulinum*, induces muscle relaxation and alleviates inflammatory pain by inhibiting acetylcholine release at presynaptic terminals and blocking pain mediators such as substance P and glutamate [16,17]. Owing to its muscle-relaxing and analgesic properties, BTX-A has been increasingly applied in for musculoskeletal pain management, particularly for myofascial pain associated with TMD. By modulating neurotransmitter and inflammatory mediator release, it provides both functional improvement and symptomatic relief [16,17].

Differential diagnoses must be thoroughly evaluated and ruled out, as many conditions may present with similar symptoms or even mimic TMD. Similar pain can originate from adjacent structures such as the eyes, ears, nose, paranasal sinuses, lymph nodes, salivary glands, or blood vessels, but is not limited to these [18,19]. Dental pain can also present with similar characteristics. Neuropathic syndromes such as various neuralgias, burning mouth syndrome, atypical facial pain, atypical odontalgia, and phantom tooth pain should also be considered. In addition, intracranial conditions (tumours, aneurysms, haemorrhages, swellings, or infections) as well as headaches (migraines, cluster headaches, tension-type headaches, or temporal arteritis) are among the most important differential diagnoses. Finally, referred pain and psychogenic causes must also be considered [18,19].

Given the complex interactions between muscle hyperactivity, myofascial pain, and structural changes within the TMJ, there is a clear need to simplify the treatment decision-making process, particularly regarding the indication for botulinum toxin therapy. This paper presents a treatment algorithm for the use of botulinum toxin in the management of TMD and bruxism. The algorithm considers anatomical, functional, and clinical variables in a manner not previously described in the literature [1]. By providing an objective framework for decision-making, the algorithm aims to optimize patient selection, reduce unnecessary interventions, and enhance treatment efficacy in TMD-related disorders. Existing studies on botulinum toxin in temporomandibular disorders and bruxism mainly focus on clinical outcomes, while evidence on structured decision support is limited. This study addresses this gap using a vignette-based pre-/post-algorithm design to assess the effect of a clinical decision algorithm on indication accuracy and clinician confidence.

Therefore, the objectives of this study were to (i) develop a concise, guideline-aligned clinical decision algorithm for BTX use in TMD and bruxism; (ii) evaluate its usability and immediate effect on diagnostic accuracy and clinician confidence; and (iii) identify priorities for future clinical validation.

## 2. Materials and Methods

This study was conducted in accordance with the principles of the Declaration of Helsinki [20]. The local ethics committee (Ethics Committee Northwest and Central Switzerland, EKNZ) was contacted. As no patients were involved and the survey was anonymous, ethical approval was not required.

Algorithm development followed a narrative synthesis of literature (PubMed and Google Scholar, inception to April 2025), prioritising systematic reviews, clinical trials, and consensus statements (e.g., AWMF). Inclusion considered diagnostic clarity, reporting quality, and clinical applicability. Key criteria included pain pattern and location, muscle activity/hypertrophy, mandibular range of motion, response to conservative therapy, and contraindications.

The initial version of the algorithm (Appendix A) was conceptualized within the framework of a master’s thesis and has since been systematically refined and further developed [1]. Its structure and content are aligned with the current guideline of the “AWMF” (Association of the Scientific Medical Societies in Germany) for the diagnosis and treatment of bruxism [10]. The final revised algorithm (Appendix B) delineates first-line conservative care and second-line escalation pathways, with BTX explicitly positioned as a second-line option following guideline-concordant therapy.

To assess the usability of the algorithm, a total of 227 dental practitioners were invited to evaluate three case vignettes, initially without and then using the provided algorithm. The case vignettes presented fictitious scenarios based on everyday practice, in which participants were asked to assess typical indications for botulinum toxin treatment. As part of a feasibility study, the case studies were initially reviewed by three experienced dentists. Their responses were not included in the final statistical evaluation. Demographic details of the pilot participants (as of 2025) were as follows:

Participant 1: Male, 54 years old (born 1971), graduated in 1999.

Participant 2: Female, 39 years old (born 1986), graduated in 2009.

Participant 3: Male, 34 years old (born 1991), graduated in 2016.

Feedback obtained from these participants is presented in Section 3.

### 2.1. Cases

The following cases were used in the survey to assess the potential role of botulinum toxin in various temporomandibular disorders.

#### 2.1.1. Case 1

A 38-year-old male patient presents with recurrent pain in the masseter muscle, especially in the mornings upon waking. He reports grinding his teeth during sleep, which his partner has observed. He has already been provided with an occlusal splint, but it has not led to satisfactory symptom improvement.

Diagnosis: Bruxism with masseter hypertrophy.

Is BTX a possible treatment option?

Yes, in therapy-resistant bruxism, targeted injections into the masseter muscle can reduce muscle activity and alleviate associated symptoms.

#### 2.1.2. Case 2

A 45-year-old female patient complains of clicking sounds in the temporomandibular joint and painful limitations during chewing. The symptoms have persisted for several years but worsened following a dental procedure. Manual therapy and physiotherapy have only provided short-term relief.

Diagnosis: Disc displacement with reduction

Is BTX a possible treatment option?

Yes, targeted injections into the masticatory muscles can reduce muscular hyperactivity and decrease joint stress, resulting in pain relief and improved mandibular mobility.

#### 2.1.3. Case 3

A 52-year-old female patient presents with bilateral temporomandibular joint pain and restricted mouth opening. She denies clenching or grinding her teeth. Imaging reveals significant degenerative changes in the TMJ, including joint space narrowing and osteoarthritic changes. Conservative therapy involving pain management and physiotherapy has been initiated.

Diagnosis: Osteoarthritis of the temporomandibular joint

Is BTX a possible treatment option?

No, botulinum toxin is not indicated for degenerative joint disease, as it does not address the underlying structural changes in the TMJ. Conservative treatment remains the primary approach, including physiotherapy, pain management, and occlusal adjustments where appropriate.

### 2.2. Data Evaluation

Participants were asked to provide their gender, age, and date of their dental degree. Responses were collected using SurveyMonkey, an online survey tool (San Mateo, CA, USA). Mandatory response fields and internal survey logic minimised missing data; all included responses were complete, and no imputation was required. Only fully paired responses across all vignettes were analysed (n = 56). No reminders were sent, and the response period was limited to five days.

This exploratory pilot study used a convenience sample to assess feasibility. Statistical analyses were performed using Microsoft Excel (version 16.95.4, Microsoft Corporation, Redmond, WA, USA). Primary outcome was paired correctness (pre- vs. post-algorithm) for BTX indications across three vignettes. Exact McNemar tests were performed for discordant pairs (b vs. c). Odds ratios (OR = b/c) and 95% confidence intervals (CIs) were obtained by transforming exact Clopper–Pearson bounds for b/(b + c). Proportions are reported with Wilson 95% CIs. “Uncertain” responses were counted as incorrect. Analyses were performed in RStudio ‘2024.12.1.563’ (“exact 2 × 2”) and validated in Excel; α = 0.05.

To differentiate between early-career and experienced clinicians, participants were categorized based on the number of years since graduation. A five-year cut-off was chosen based on the theory of deliberate practice proposed by Chase and Simon [21], which suggests that expert performance typically requires approximately 10,000 h of structured practice. To align the experience split with empirical evidence in dentistry, we classified respondents into ≤5 years versus >5 years of clinical practice. This threshold is supported by studies showing that dentists with approximately 5 or more years of practice demonstrate higher diagnostic reproducibility and accuracy in radiographic caries detection than final year students [22] and that diagnostic ability differs significantly when <5-year dentists are contrasted with those >5 years in oral pathology recognition [23].

## 3. Results

### 3.1. Participant Characteristics

A total of 227 dentists were contacted, of whom 103 (57.3%) responded to the survey. Among these, 59 participants (26%) completed the questionnaire in full. Three participants were excluded from the analysis because they held both a dental and a medical degree. The final sample therefore included 56 (24.7%) dentists with dental degrees only. Of the included participants, 57.1% (n = 32) were male and 42.9% (n = 24) were female. The mean age of participants was 34.5 years (±10.6 years; range 23 to 61 years). On average, participants had graduated from dental school 9.5 years ago (minimum 1 year, maximum 36 years). Half of the dentists (50%) had graduated within the past five years. Only 7.1% of respondents reported administering botulinum toxin in their clinical practice. All participants were professionally active in Switzerland at the time of the survey.

### 3.2. Pilot Study Feedback

Feedback from the pilot study led to several key adjustments to the main survey:

Participant 1 reported difficulty reading the algorithm due to the small font size in the survey interface. As a result, the font size was increased, and the visual formatting was improved for better readability in the main study. He also suggested displaying the algorithm in a separate browser window for better readability.

Participant 2 recommended explicitly instructing participants not to use external resources, such as internet searches, to ensure that responses reflected their clinical knowledge and judgment. This suggestion was not implemented, as the survey focused on clinical judgments based on scenario evaluations rather than pure knowledge-based questions.

Participant 3 proposed aligning the survey’s treatment options with those presented in the algorithm. Specifically, they recommended combining behavioural therapy and psychological coaching into a single selection option to ensure consistency. Additionally, they recommended fully translating the algorithm into German, given that the survey targeted dentists practicing in the German-speaking part of Switzerland.

All recommendations were implemented except for two: the suggestion to prohibit external resources and the recommendation to display the algorithm in a separate browser window, both of which were not supported by the SurveyMonkey platform.

These modifications improved clarity, usability, and consistency for the participants in the main study.

### 3.3. First- and Second-Line Treatment Decisions

As shown in the Table A1: Changes in first- and second-line treatment decisions. Before vs. after studying the algorithm. “↑” = increase, “ ↓” = decrease and “—“ for no change.” in Appendix C, application of the treatment algorithm led to substantial changes in clinical decision-making, particularly in Cases 1 and 2.

In Case 1, more participants selected cold/heat therapy, muscle relaxants, analgesics, splint therapy, and occlusal adjustment after using the algorithm. Minor or no changes were observed for physiotherapy, psychological therapy/stress management, and corticosteroids.

Similarly, in Case 2, arthrocentesis, cold/heat therapy, and muscle relaxants were chosen more often following algorithm use, whereas splint therapy and occlusal adjustment saw significant declines. Corticosteroid use increased slightly, reaching trend-level significance, while physiotherapy, analgesics, and psychological therapy/stress management did not change significantly.

In Case 3, algorithm use was associated with increases in the selection of arthrocentesis, corticosteroids, cold/heat therapy, muscle relaxants, and psychological therapy/stress management. Conversely, the use of analgesics and physiotherapy declined significantly. Other treatment options, such as splint therapy and occlusal adjustment, remained unchanged.

### 3.4. Botulinum Toxin Decision Accuracy and Confidence

The application of the algorithm improved both the accuracy and confidence of decision-making regarding botulinum toxin use as summarized in Table 1. In Case 1, the proportion of correct decisions increased from 62.5% to 87.5% (*p* = 0.0013). In Case 2, accuracy rose sharply from 14.3% to 87.5% (*p* < 0.0001). In contrast, no significant change was observed in Case 3 (44.6% vs. 46.4%; *p* = 1.000), consistent with the fact that botulinum toxin was not indicated in that scenario.

**Figure 1:**
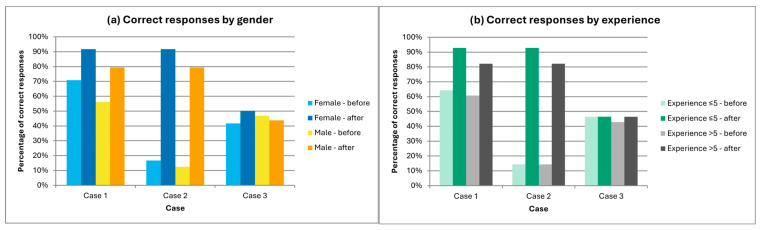
Survey outcomes by clinical case and correct responses. (**a**) Correct responses by gender. Bars display the percentage of correct decisions for each case, separated by female and male participants, before and after algorithm use. (**b**) Correct responses by clinical experience. Bars display the percentage of correct decisions for each case, separated by dentists with ≤5 years and >5 years of experience, before and after algorithm use.

As illustrated in Figure 1, subgroup analyses revealed statistically significant improvements in Cases 1 and 2 for both male and female participants. Among dentists with less professional experience, decision accuracy improved significantly in both Case 1 (*p* = 0.021, OR = 0.111 (0.003–0.802)) and Case 2 (*p* < 0.001, OR = 0.023 (0–0.183); *p* = 1 for Case 3). For more experienced dentists, statistical significance was achieved only in Case 2 (*p* < 0.001, OR = 0.026 (0–0.214); *p* = 0.070, OR = 0.143 (0.003–1.111) for Case 1 and *p* = 1 for Case 3).

The proportion of “uncertain” responses declined significantly after algorithm application:

Among less experienced dentists, uncertainty decreased in all three cases (Case 1: *p* = 0.0125; Case 2: *p* < 0.0001; Case 3: *p* = 0.0020).

Among more experienced dentists, the decline of uncertainty was significant in Case 2 (*p* = 0.0010) and Case 3 (*p* = 0.0078), but not in Case 1 (*p* = 0.4531).

No significant gender-based differences were observed in uncertainty rates before or after algorithm use. (before: *p* = 0.123, χ^2^ = 2.38; after: *p* = 0.153, χ^2^ = 2.04).

### 3.5. Usefulness of the Algorithm

Overall, 78.6% of participants considered the algorithm helpful for clinical decision-making. No significant differences in usefulness ratings were observed based on gender (*p* = 0.372) or professional experience (*p* = 1).

### 3.6. Analgesic Selection Patterns

Among participants who selected analgesics in Cases 1–3, no significant differences were found in the specific substances chosen (paracetamol, NSAIDs, Novalgin, or others). NSAIDs were the most frequently chosen analgesics across all cases. A chi-square test indicated no statistically significant variation in selection patterns (χ^2^ = 3.4, *p* = 0.493), suggesting consistent analgesic preferences regardless of the clinical scenario.

## 4. Discussion

The COVID-19 pandemic has been associated with a notable increase in TMD cases and bruxism, largely attributed to heightened stress and anxiety levels. This trend underscores the need for effective treatment strategies [24,25]. To assist dental practitioners in determining when botulinum toxin is an appropriate treatment option, we developed a concise algorithm designed for easy implementation in clinical practice Based on participant feedback, we have made further adjustments to the algorithm. The final version is presented in Appendix B.

Assessing the algorithm’s applicability was a key objective of this study. With overall response rates exceeding 50% and 24.7% for complete surveys from participants holding only a dental degree, participation was deemed sufficient. The surveyed dentists were either currently practicing or had previously practiced in proximity to the authors, which likely contributed to these relatively high rates. In contrast, other online studies have reported substantially lower response levels [26,27]. One clear advantage of online surveys over postal surveys is the absence of mailing costs. Additionally, compared with telephone interviews, online surveys enable participants to consult the algorithm directly while completing their responses. Furthermore, for the survey administrators, sending emails requires considerably less time and effort than conducting multiple phone calls [28].

### 4.1. Algorithm Design Considerations

To ensure clarity and practical applicability, the algorithm was deliberately restricted to essential decision pathways, emphasizing core clinical logic rather than exhaustive detail. Although incorporating additional interdependencies or bidirectional links between decision boxes could capture greater nuance, these elements were intentionally minimized to maintain accessibility and ease of use. Following response analysis, ambiguity between disc displacement and osteoarthritis was removed, and BTX was explicitly confined to refractory myogenous pain after guideline-concordant therapy. The result is a pragmatic, reproducible instrument intended for streamlined use in daily practice, without purporting to address every possible clinical scenario.

### 4.2. Diagnostic and Treatment Options for TMD

For many years, the Helkimo Index [29] and the Research Diagnostic Criteria for Temporomandibular Disorders [30] were the standard diagnostic tools, as noted by Kapos et al. These frameworks later evolved into the Diagnostic Criteria for Temporomandibular Disorders [31], a validated and widely recognized methodology for assessing TMD. This standardized method enables systematic evaluation of relevant anatomical structures, psychosocial factors, and comorbidities, while ensuring reproducibility and accurately reflecting patients’ real-world symptoms. Additionally, it is recommended to assess pain areas using the TMD Pain Screener or other validated screening tools [7].

Diagnosis is structured along two axes. Axis I focuses on the 12 most common TMD diagnoses, six of which are pain-related: myalgia (local myalgia, myofascial pain, and myofascial pain with referral), arthralgia, headache attributed to TMD, four types of disc displacement, degenerative joint disease, and subluxation [7,31,32]. Axis II addresses psychosocial factors, comorbidities, and other patient characteristics [7].

Kapos et al. recommend a gradual, stepwise treatment approach, reserving invasive and irreversible therapies for carefully selected cases [7].

TMD encompasses a range of treatment approaches, including pharmacological interventions, behavioural therapies, and mechanical therapies. The selection of an appropriate therapy depends on the underlying cause, symptom severity, and individual patient-specific factors. Given the bidirectional interaction between stress, sleep disturbances, and masticatory muscle activity, psychological assessment and adjunctive behavioural strategies may enhance BTX outcomes when indicated [33] because psychological factors such as stress, anxiety, depression, and catastrophizing play a key role in the onset, severity, and persistence of temporomandibular disorders (TMDs) [34,35,36,37]. These factors not only exacerbate pain and increase disability but may also influence neuromuscular activity in the masticatory system: for example, chronic stress has been associated with increased temporalis muscle episodes during sleep [33]. Furthermore, psychosocial disorders and sleep disturbances are increasingly recognised to modulate masticatory muscle activity and thus may affect the effectiveness of botulinum toxin (BTX-A) therapy for TMD [35,38]. By reducing muscle hyperactivity and blocking pain neurotransmitter release, BTX-A offers therapeutic benefit, yet its outcomes may be modulated by underlying psychological and behavioural factors [22,35]. Incorporating psychological assessment and interventions alongside BTX-A-based treatment may therefore enhance pain control, reduce functional impairment, and improve overall patient well-being [35,36,37,38].

Understanding these treatment modalities is essential for integrating botulinum toxin into a structured algorithm for TMD management. Key decision parameters for BTX consideration include: a primary myogenous pain phenotype localized to masticatory muscles; clinical or imaging evidence of muscle hyperactivity or hypertrophy; presence of clinically relevant bruxism; and insufficient improvement following structured first-line conservative therapy. These criteria are consistent with current literature supporting BTX use in refractory myogenous TMD following conservative therapy failure [17,39,40]. For acute symptoms, anti-inflammatory medications can be administered either topically or systemically; however, their use should be limited to a short duration. In cases of myogenic pain, tricyclic antidepressants (TCAs) have demonstrated efficacy [1,2,3]. TCAs act by binding to adrenergic, histaminergic, and cholinergic receptors, thereby inhibiting the reuptake of neurotransmitters [41]. Nonetheless, TCAs are associated with anticholinergic side effects, including dry mouth, urinary retention, and, particularly in elderly patients, disturbances in cardiac conduction and delirium [41].

A commonly recommended method involves educating patients on self-monitoring and maintaining a relaxed mandibular posture [42]. Furthermore, patients should be advised to eliminate parafunctional habits, including unconscious clenching, forward displacement of the mandible, and cheek or lip biting. Visual reminders, like adhesive dots placed on frequently viewed surfaces (e.g., phone screens, watches, refrigerators), can help patients to keep a relaxed jaw posture. Stretching exercises and circular massage techniques can also help promote relaxation [1,2,3].

Occlusal splints are frequently used in TMD therapy to reduce habits such as clenching and grinding [1,2,3]. Clinically, some patients continue to grind and clench while wearing the splint, sometimes altering its surface to achieve even occlusion [43].

An alternative is the stabilization (Michigan) splint, which provides a wide range of therapeutic effects. By altering the mandibular position, it activates different motor units in the masticatory muscles. This redistribution can lessen the strain on overworked motor units, thereby reducing pain. Also, the psychological impact of splint use, although not extensively evidenced, should not be underestimated. For many patients, the splint acts as a psychological cue, reducing nocturnal bruxism and protecting the TMJ and masticatory muscles from excessive stress. The aim of a Michigan splint is to establish optimal static and dynamic occlusion while maintaining a physiologically stable mandibular position [43].

TMD patients frequently report elevated stress levels, making stress management an essential component of treatment. Patients should be encouraged to implement behavioural changes and engage in regular physical activity to reduce stress [1,2,3]. Additionally, thermotherapy (the application of heat and cold) can help relax muscles and reduce discomfort [44].

A structured algorithm for TMD treatment must include a multimodal approach, combining pharmacological, behavioural, mechanical, and alternative therapies. While botulinum toxin represents a promising option for persistent muscular hyperactivity, its use should be carefully integrated into the broader treatment spectrum of TMD. Patients with primary myofascial pain or stress-related parafunctional habits may benefit more from relaxation techniques and occlusal splints before considering botulinum toxin. However, individuals with refractory bruxism and masseter hypertrophy may be suitable candidates for targeted botulinum toxin injections within a stepwise treatment plan.

In support of this tiered approach, the survey revealed that the algorithm significantly influenced treatment patterns across all three clinical cases. In Case 1, participants showed statistically significant increases in the selection of cold/heat therapy, muscle relaxants, analgesics, splint therapy, and occlusal adjustment. In Case 2, a more complex clinical presentation, the algorithm prompted an increase in arthrocentesis, cold/heat therapy, muscle relaxants, and corticosteroids, while decreasing splint therapy and occlusal adjustment. Case 3 followed a similar pattern, with significant increases in arthrocentesis, corticosteroids, muscle relaxants, and stress management interventions, accompanied by a decrease in the use of physiotherapy and analgesics. These findings confirm the algorithm’s capacity to promote context-specific, evidence-based decision-making across varying TMD presentations.

Across all three clinical cases, no statistically significant interaction was observed between professional experience (less- vs. more-experienced dentists) and use of the algorithm regarding first- and second-line treatment decisions. Although some first- and second-line options showed numerically greater changes in one group (e.g., cold/heat therapy in Case 1), none of the between-group differences reached statistical significance (all *p* > 0.25). This suggests that the algorithm had a comparable impact on first- and second-line therapeutic choices, regardless of the dentists’ level of clinical experience.

By systematically assessing each case according to symptomatology, contributing factors, and previous treatment responses, an algorithm can optimize patient selection, reduce unnecessary interventions, and improve therapeutic outcomes in TMD management.

The use of botulinum toxin for TMD treatment aims to reduce bruxism episodes and associated mechanical forces by inhibiting the activity of the masticatory muscles. This approach helps protect teeth and alleviate pain [45]. Additionally, studies have demonstrated that botulinum toxin injections may reduce headache intensity associated with TMD [46]. The peak therapeutic effect of botulinum toxin has been reported at 5 to 6 weeks post-injection [47], whereas another study observed greater clinical benefit approximately 10 weeks after injection [2].

Botulinum toxin is typically administered into the masseter muscle and, in some cases, the temporalis muscle. Injection sites vary across studies. Pihut et al. targeted the region with the greatest overlap of the masseter muscle bellies for injection [46]. Conelly et al. administered three injections in the lower masseter and two in the anterosuperior portion of the temporalis muscle [2]. A review by De la Torre Canales et al. analysed various studies, most of which reported three injections in the masseter. In comparison, one study used four injections in the masseter and three in the temporalis [48].

When administering botulinum toxin, it is recommended to begin with the lowest effective dose. Dosages vary depending on muscle mass, ranging from 8 to 100 units for the masseter and 0 to 25 units for the temporalis [48,49]. Dose selection should be based on muscle mass, as excessive amounts may result in undesired diffusion beyond the target area [50,51].

The therapeutic effect of botulinum toxin diminishes over time, necessitating repeat injections for sustained benefit. With successive treatments, the duration of effect may increase, allowing for extended treatment intervals [52]. One study reported a significant reduction in masseter volume two weeks after injection, likely explaining the observed decrease in muscle activity and the prolonged therapeutic effects. Accordingly, dosage adjustments may be required over time [53]. For repeated injections, the lowest effective dose should be administered at the longest clinically acceptable interval. The total amount of botulinum toxin depends on the specific muscle being targeted and the number of injection sites administered in a single session. In paediatric patients, botulinum toxin treatment is either avoided or used at substantially lower doses [1,54].

The integration of botulinum toxin into a TMD treatment algorithm requires careful consideration of dosage, muscle targeting, and long-term treatment planning. By selecting appropriate patients, optimizing injection sites, and adjusting dosages based on clinical response, botulinum toxin can be an effective component of a structured, stepwise approach to TMD and bruxism management.

Survey results confirmed the algorithm’s value in guiding decisions regarding botulinum toxin. Correct decisions increased significantly in Case 1 (62.5% → 87.5%; *p* = 0.0013) and Case 2 (14.3% → 87.5%; *p* < 0.0001), whereas Case 3 (where botulinum toxin was not indicated) showed no significant change. The number of uncertain responses decreased notably, particularly among less-experienced dentists, highlighting the algorithm’s educational value. Subgroup analysis further revealed significant improvements in both genders and across experience levels, with the largest gains among less-experienced clinicians in Cases 1 and 2. These findings support the notion that structured tools can bridge knowledge gaps and build confidence in clinical decision-making.

Similarly, no statistically significant interaction was found between experience level and changes in decision accuracy for botulinum toxin use. Both less- and more-experienced dentists improved notably in Cases 1 and 2, with statistical significance achieved only in Case 2 for both groups. Likewise, no significant interaction was observed between experience level and decision accuracy. Both groups, less- and more-experienced, improved in Cases 1 and 2, with significance reached only in Case 2 for both. These findings suggest that the algorithm effectively enhanced clinical decision-making across different levels of professional experience.

Despite differences in clinical scenarios, the distribution of selected analgesics remained largely consistent across all three cases, with NSAIDs being the most frequently chosen. The lack of statistically significant variation (*p* = 0.408) indicates a reliance on routine prescribing habits rather than scenario-specific considerations.

While the algorithm generally improved clinical decision-making, in Case 3 (osteoarthritis) it led some participants to incorrect conclusions: nine individuals who had initially answered “no” to botulinum toxin changed their response to “yes” after using the algorithm. This outcome was likely influenced by a bidirectional arrow between disc displacement and osteoarthritis, which may have implied overlapping treatment indications. In response, the algorithm was revised by removing the arrow and adding treatment labels such as first-line, second-line, and off-label use to improve clarity and better guide clinical priorities.

Current guidelines are limited to aesthetic botulinum toxin therapy [55], bruxism therapy [10], and TMD treatment using occlusal splint therapy [56]. In 2022, the “German Society for Functional Diagnostics and Therapy” issued a scientific statement on the treatment of craniomandibular dysfunctions. It outlines dental functional therapy as the initial step in TMD treatment, utilizing removable splints, including relaxation splints, reflex splints, positioning splints, and occlusal splints. As a second step, the statement recommends definitive dental measures in the form of irreversible occlusal interventions. Orthodontic treatments, surgical therapies, physiotherapy, physical-medical, psychosomatic and psychological therapies, and pharmacological treatments are considered to be on the same level as dental therapies [57]. This hierarchy is reflected in the developed algorithm, demonstrating that the appropriate therapy selection must be made from equally ranked options for each patient. Botulinum toxin injections into the masticatory muscles are recommended only for persistent symptoms following conservative therapy and require thorough patient education. In Switzerland, botulinum toxin injections into the masticatory muscles are not covered by health insurance and are considered an off-label indication. This gradation is also reflected in the algorithm as an off-label indication [57].

### 4.3. Risks and Considerations of Botulinum Toxin Treatment

The use of botulinum toxin for the treatment of masticatory muscle disorders is considered an off-label therapy. Comprehensive informed consent and cost discussion are mandatory; BTX is considered second-line after conservative therapy. While generally well tolerated, it carries certain risks, particularly in patients with underlying neuromuscular disorders such as myasthenia gravis, Eaton-Lambert syndrome, peripheral motor neuropathies (e.g., amyotrophic lateral sclerosis (ALS)), and neurological diseases such as multiple sclerosis (MS). These patients may exhibit increased sensitivity to botulinum toxin, even at therapeutic doses [54].

Cardiovascular side effects such as arrhythmias or myocardial infarction, as well as new or recurrent epileptic seizures, have also been documented [54].

Hypersensitivity reactions, although rare, may include anaphylaxis, serum sickness, urticaria, soft tissue edema, and dyspnea. Local adverse effects at the injection site can include infection, inflammation, paraesthesia, hypoesthesia, tenderness, swelling, bruising, pain, or erythema. Vasovagal reactions triggered by the injection procedure itself are also possible [54].

Overdosing can cause systemic muscle weakness, including in areas far from the injection site, potentially leading to dysphagia, pneumonia, or widespread weakness [54].

When botulinum toxin A is used for treating masseter hypertrophy, patients should be informed about potential aesthetic changes. As the toxin causes muscle atrophy, facial contours may become less angular, and the jawline less defined. While this effect often may be desirable in women with naturally triangular faces, it may be less favourable for male patients who wish to maintain a prominent jawline [58].

These considerations highlight the importance of careful patient selection, comprehensive pre-treatment counselling, and precise injection techniques when integrating botulinum toxin into an algorithm for TMD management.

Other studies have also investigated the applicability of algorithms in supporting decision-making and ensuring accurate diagnostics.

### 4.4. Structured Algorithm Usage and Limitations

Vivaqua et al. developed a structured algorithm for differentiating jaw pain in divers, designed for use by diving physicians and other medical professionals without specialized dental or maxillofacial expertise. The authors noted that jaw pain in divers can arise from various causes, including poorly fitting mouthpieces that induce jaw misalignment. Application of their algorithm enabled all participating diving physicians to successfully diagnose the presented case studies, highlighting its practical utility [59].

Despite the demonstrated benefits of such tools, access to or awareness of diagnostic algorithms often remain limited within certain departments or clinical settings. Sommacal et al. investigated this issue by assessing the availability and use of dental and maxillofacial emergency algorithms across Swiss emergency departments [60].

Following survey completion, several refinements were implemented to improve the algorithm’s structure and clinical clarity. The version used during data collection is provided in Appendix A, whereas a revised final version, intended for future clinical application, is included in Appendix B. Maintaining a concise format, rather than expanding it further, ensures its practicality for daily use, allowing it to remain easily accessible and printable [60]. Its primary purpose is to support dentists in determining whether botulinum toxin is an appropriate treatment option in specific cases.

A potential limitation of this study is the relatively small number of dentists involved in evaluating the algorithm. This study represents a pilot feasibility evaluation. Therefore, it used a convenience sample to assess feasibility; future studies will include formal power calculations based on effect size estimates.

The limited sample size (n = 56) constrains generalisability; multicentre and cross-system validation studies are planned to confirm robustness and scalability. Also, the short recruitment window may introduce a self-selection bias. Future work will also assess inter-rater reliability, objective clinical outcomes, and patient-reported measures including pain reduction, functional improvement, and satisfaction. Other important items such as psychosocial comorbidities, chronic pain phenotypes, and combined TMD presentations should also be considered to better reflect real-world complexity.

## 5. Conclusions

This study demonstrates that a structured, clinically oriented algorithm can significantly enhance dentists’ decision-making in the management of craniomandibular dysfunction (CMD), particularly regarding the use of botulinum toxin and the selection of first- and second-line therapies. The algorithm was effective across varying levels of clinical experience, enhancing both decision accuracy and practitioner confidence. While it promoted consistent therapeutic choices, certain areas, such as analgesic selection, remained largely unaffected, reflecting the persistence of habitual prescribing patterns. Minor misinterpretations in one clinical case prompted targeted revisions of the algorithm, including clearer treatment hierarchies and the removal of ambiguous visual elements. Overall, the algorithm shows strong potential as a practical decision-support tool in dental practice and may contribute to more standardized, evidence-based management of TMD.

## Figures and Tables

**Table 1:** Accuracy of clinical decisions regarding botulinum toxin use. Before vs. after studying the algorithm.

Case	Time Point	Correct (%)	Incorrect (%)	Uncertain (%)	OR (95% CI)	*p*-Value *
1	pre	35 (62.5%)	4 (7.1%)	17 (30.4%)	0.125	0.0013
	post	49 (87.5%)	2 (3.6%)	5 (8.9%)	(0.014–0.532)	
2	pre	8 (14.3%)	21 (37.5%)	27 (48.2%)	0.012	<0.0001
	post	49 (87.5%)	3 (5.4%)	4 (7.1%)	(0.000–0.094)	
3	pre	25 (44.6%)	8 (14.3%)	23 (41.1%)	0.9	1.000
	post	26 (46.4%)	25 (44.6%)	5 (8.9%)	(0.324–2.464)	

* Based on McNemar tests with “uncertain” and “incorrect” answers combined in one category. N = 56 in all cases.

**Figure A1:**
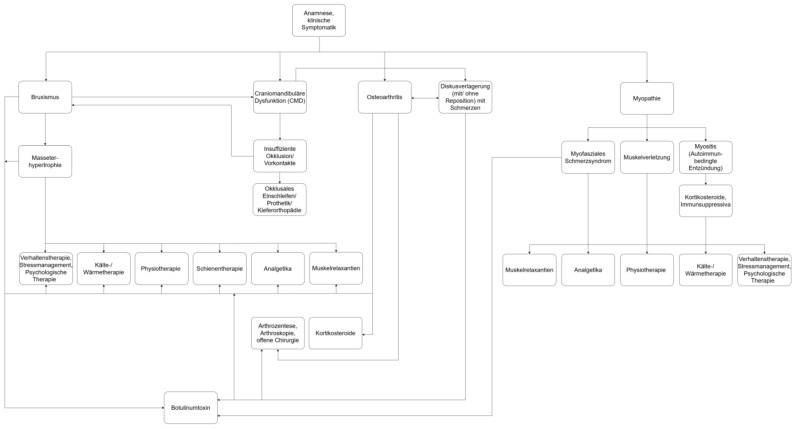
Decision algorithm for CMD management. Overview of the decision algorithm for CMD management as used the survey. Abbreviation: CMD = craniomandibular dysfunction.

**Figure A2:**
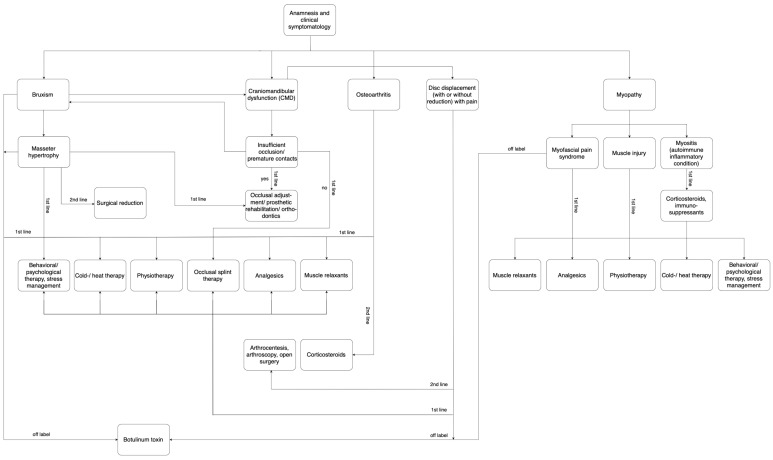
Decision algorithm for CMD management. Overview of the updated decision algorithm for CMD management. The arrows represent treatment progression and show the way from anamnesis and clinical symptomatology to the appropriate therapy. The first grade includes different diagnoses, and the subgroups are outlined in the second grade. Third and fourth grade are reached via 1st line arrows, which indicate first treatment possibilities, and 2nd line shows the following treatment options. The last grade contains botulinum toxin and is reached via arrows that indicate the off-label indication that needs to be considered. Abbreviations: CMD = craniomandibular dysfunction; BTX = Botulinum toxin.

## Data Availability

The datasets generated and analysed during the present study are available from the corresponding author upon reasonable request.

## Abbreviations

The following abbreviations are used in this manuscript:

AB	Awake bruxism
BTX	Botulinum toxin
CMD	Craniomandibular Dysfunction
MAP	Myoarthropathic complaints (Swiss term)
SB	Sleep bruxism
TCAs	Tricyclic antidepressants
TMD	Temporomandibular disorders
TMJ	Temporomandibular joint

## Appendix C

**Table A1:** Changes in first- and second-line treatment decisions. Before vs. after studying the algorithm. “↑” = increase, “ ↓” = decrease and “—“ for no change.

Case	Treatment Option	Pre n	Post n	*p*-Value	Direction
1	Cold/heat therapy	16	45	<0.00000001	↑
1	Muscle relaxants	20	44	<0.000001	↑
1	Analgesics	31	45	0.00012	↑
1	Splint therapy	33	45	0.00049	↑
1	Occlusal adjustment	4	11	0.0156	↑
1	Physiotherapy	49	51	0.4531	↑
1	Psychological therapy/stress management	49	49	1.000	—
1	Corticosteroids	2	3	0.3173	↑
2	Arthrocentesis	6	30	<0.000001	↑
2	Cold/heat therapy	12	22	0.00195	↑
2	Muscle relaxants	11	21	0.00195	↑
2	Corticosteroids	6	11	0.0625	↑ (trend)
2	Splint therapy	37	28	0.00391	↓
2	Occlusal adjustment	14	5	0.00391	↓
2	Physiotherapy	34	29	0.1573	↓
2	Analgesics	29	25	0.4241	↓
2	Psychological therapy/stress management	26	26	1.000	—
3	Arthrocentesis	21	40	0.000004	↑
3	Corticosteroids	27	42	0.000061	↑
3	Cold/heat therapy	10	23	0.000244	↑
3	Muscle relaxants	9	20	0.000977	↑
3	Psychological therapy/stress management	16	24	0.007812	↑
3	Analgesics	36	24	0.000488	↓
3	Physiotherapy	34	26	0.007812	↓
3	Splint therapy	26	26	1.000	—
3	Occlusal adjustment	3	4	1.000	↑
